# Largemouth bass (*Micropterus salmoides*) exhibited better growth potential after adaptation to dietary cottonseed protein concentrate inclusion but experienced higher inflammatory risk during bacterial infection

**DOI:** 10.3389/fimmu.2022.997985

**Published:** 2022-09-15

**Authors:** Mengya Wang, Zhenwei Chen, Yunhua Wang, Jiahong Zou, Shuaitong Li, Xiaolong Guo, Jian Gao, Qingchao Wang

**Affiliations:** Department of Aquatic Animal Medicine, College of Fisheries, Huazhong Agricultural University, Wuhan, China

**Keywords:** cottonseed protein concentrate, largemouth bass, liver health, Nocardia seriolae, inflammation

## Abstract

Cottonseed protein concentrate (CPC) has been proven to partially replace fishmeal without adverse effects on fish growth performance, while little information is known about the effects on liver health during bacterial infection. In the present study, 15% CPC was included into the diet of juvenile largemouth bass (32.12 ± 0.09g) to replace fishmeal for 8 weeks, with fish growth potential and hepatic inflammatory responses during *Nocardia seriolae* (*N. seriolae*) infection systemically evaluated. After adaptation to dietary CPC inclusion, largemouth bass even exhibited better growth potential with higher SGR and WGR during the last three weeks of whole feeding trial, which was accompanied with higher phosphorylation level of TOR signaling and higher mRNA expression level of *myogenin* (*myog*). At the end of 8-weeks feeding trial, the histological structure of largemouth bass liver was not significantly affected by dietary CPC inclusion, accompanied with the similar expression level of genes involved in innate and adaptive immunity and comparable abundance of T cells in bass liver. *N.seriolae* infection induced the pathological changes of bass liver, while such hepatic changes were more serious in CPC group than that in FM group. Additionally, RT-qPCR results also suggested that largemouth bass fed with CPC experienced much higher inflammatory potential both in liver and gill during *N. seriolae* infection, which was accompanied with higher expression level of genes involved in pyroptosis. Therefore, this study demonstrated that the application of CPC in largemouth bass diet should be careful, which may induce higher inflammatory potential during *N. seriolae* infection.

## Introduction

Aquaculture supplies plenty of high protein food for human beings and has become the fastest animal-protein producing industry among all animal husbandry in the past 30 years (1). Feed expenses have always been the main cost for fish farmers, and fish meal has been traditionally regarded as the highest quality protein raw material for aquafeed due to its high-quality amino acid ratio, good palatability and perfect digestibility (2). However, global fishmeal production cannot reach a stable increase like the aquaculture production and the fishmeal price has significantly increased year by year (3). It is important to search for alternative protein sources to reduce fishmeal utilization in fish diet, and soybean meal has been widely used in advanced aquafeed because of its high protein content and balanced amino acid composition (4). In China and other main aquaculture countries, the soybean self-production capacity is relatively weak and the global soybean application has been pledged to be restricted which are in the expense of deforestation. Thus, it has become an industry consensus to develop new raw materials and reduce the use of fish meal and soybean meal in aquafeed. Many protein sources have also been tested in fish diet, including rapeseed meal, peanut meal and many others (5). However, the main constraint for these alternative sources remains in their much lower production than soybean meal. Cotton is one of the most important crops in the world, with large output but low production cost, which makes the price of cotton products relatively low (6). During the process of cotton production, a large number of by-products such as cottonseed can be produced, and developing them as feed protein raw materials not only has important economic value, but also has important ecological significance for reducing carbon emissions. With cottonseed as the basic raw material, cottonseed protein concentrate (CPC) is produced through low-temperature oil extraction, removal of gossypol and other process stages, based on the “liquid-liquid-solid” extraction technology production processes. The crude protein level of CPC could reach up to 60%, which is rich in arginine, leucine, phenylalanine and other essential fish amino acids. Additionally, some water-soluble non-starch polysaccharides, oligosaccharides, and anti-nutritional factors such as phenol and tannin has been removed to significantly improve its nutritional value and palatability (7). CPC has been proved to effectively replace partial FM without adverse effects on growth of silver sillago (*Sillago sihama*), golden pompano (*Trachinotus ovatus*), hybrid grouper (*Mycteroperca tigris × Epinephelus lanceolatus*) (8), red drum (*Sciaenops ocellatus*) (9), and rainbow trout (*Oncorhynchus mykiss*) (10).

With the urbanization, the demand for high-nutritional value and bone-less fish species is significantly increased in many countries including China (11). Largemouth bass (*Micropterus salmoides*), a carnivorous fish species originating from Canada and America, has been widely cultured all over the world. Ever since it was introduced to China in 1980s, the aquaculture production of largemouth bass has significantly increased year by year and has reached 619, 519 tons in 2020 (12). During aquaculture, the liver health of largemouth bass has received much attention. Liver structure was reported to be significantly affected by dietary glucose level (13). Additionally, *N. seriolae* infection, which is rather common in largemouth bass aquaculture, also caused serious hepatic damage (14). In fact, liver receives dual blood supply from the portal vein and hepatic artery, and both acts as the metabolic center and performs immune-regulatory roles (15). The liver is constantly bombarded by a stream of dietary and commensal bacterial products with inflammatory potential even in healthy individuals, which results in persistent, regulated inflammation (16). Such inflammation creates the potential for excessive immune activation during challenge by pathogens or tissue damage, which could be resolved during appropriate immune activation to maintain liver homeostasis. Failure to clear ‘dangerous’ stimuli or regulate appropriately activated immune mechanisms leads to chronic pathological inflammation and disrupted tissue homeostasis characterized by the progressive development of fibrosis, cirrhosis and eventual liver failure (17). Especially, during fishmeal replacement by plant proteins, the existing anti-nutritional factors in plant sources would be transferred to liver, which may cause continual inflammatory stimulus to liver and even immune fatigue in liver. When fish was exposed to pathogenic infection, the chronic pathological inflammation may occur which results in the liver failure. In largemouth bass, CPC has been reported to replace fishmeal at different ratios in different studies without adverse effects on fish growth performance (18–20). However, the immune status and tissue health have been rarely evaluated, especially the immune status during bacterial challenge.

As mentioned above, *N. seriolae* infection seriously affected the health of largemouth bass and resulted in the damage of internal organs including liver and eventual high mortality (14). *N. seriolae* infection has been reported to induce high inflammatory responses in bass liver (21) while little information is known about the metabolic control of such inflammatory responses in liver. Considering the high production of CPC and its application potential in diet for largemouth bass, it is important to study the influences of CPC inclusion on liver structure and function, especially the inflammatory responses during bacterial infection. Thus, the influences of dietary cottonseed protein concentrate replacing fishmeal on growth potential, liver health status and challenge resistance of largemouth bass (*Micropterus salmoides*) were systemically evaluated in the present study. To our acknowledge, this is the first study to evaluate the effects of CPC on fish hepatic inflammatory responses during nocardiosis.

## Materials and methods

### Ethics statement

All the fish care and experimental procedures were approved by the Animal Experiment Committee of Huazhong Agricultural University (permit number HZAUFI-2016-007).

### Feed formulation, fish husbandry and sampling

Two isoproteic (50%) and isolipidic (12.5%) diets were formulated based on fish meal (FM) or cottonseed protein concentrate (CPC), respectively (**Table 1**). In order to balance the amino acid composition, krill meal, blood cells, wheat gluten and corn gluten meal were also included in each diet. Attractant (betaine) and taurine were also added to improve the food palatability.

**Table 1:** Formulation and proximate chemical composition of the tested diets (% dry matter).

	FM	CPC
Fish meal	48	35
Cottonseed protein concentrate	0	15
Krill meal	3	3
Wheat gluten	4	4
Blood meal	2	2
Corn gluten meal	5	5
Soybean meal	0	0
Wheat meal	6	6
Cassava starch	6	6
Beer yeast	2.5	2.5
Bentonite	13.1	9.8
Fish oil	4	5
Lecithin high potency	2	2
Vitamin mineral premix	2	2
Alanine	1.5	0.5
Taurine	0	1
Betaine	0.5	0.5
Choline chloride	0.25	0.25
Calcium bis	0	0.3
Calcium Propionate	0.1	0.1
Ethoxyquin	0.05	0.05
Proximate composition		
Crude protein	47.15	47.35
Crude fat	10.68	10.86

Juvenile largemouth bass were acclimated to our laboratory conditions and experimental feeds for two weeks before being randomly distributed into fibre-glass tanks of 400-L capacity. Experiment was done in wet lab of College of Fisheries, Huazhong Agricultural University. Fish with initial weight of 32.12 ± 0.09 g were distributed into 6 experimental tanks (400-L) which were connected to a circulating water system. The water parameters in circulating water system were kept constant with water flow at 0.5 L/min, water temperature at 26 ± 2 °C, and oxygen content of outlet water at over 85% saturation. Day length and dark ranged over the course of the trial following natural changes. Each diet was randomly allocated to triplicate groups of fish for 8 weeks. Feed was offered by hand to apparent satiety twice per day, with feed consumption was recorded. After 2 weeks, 5 weeks and 8 weeks, all fish were counted and group-weighed under moderate anaesthesia (3-aminobenzoic acid ethyl ester, MS 222; 100 μg/mL) for growth parameters calculation.

At the end of the feeding trial, total fish number and body weight in each tank were counted and measured after being anesthetized by MS222, respectively. Blood samples were collected from the caudal vessels of 9 fish from each group with heparinized syringe. Then fish were perfused with PBS to discard the inference of blood on liver and gill immune responses. Liver and gill tissues were separated, with partial segments stored in paraformaldehyde. Other tissue samples (serum, gill, liver and others) were immediately frozen in liquid nitrogen and then stored at -80°C for further assay.

### Bacterial challenge

*N. seriolae* strain HG2101, was isolated from an infected largemouth bass and preserved in our laboratory. The whole genome sequence has been sequenced by our laboratory to confirm the accurate (unpublished). Before the formal experiment, a preliminary study was conducted to determine the LD50 of this bacterial strain in largemouth bass. Thus the infection concentration in the formal experiment was finally determined as 1.0 × 10^7^ CFU/mL. Before bacterial challenge experiment, the *N. seriolae* was taken out from −80°C refrigerator and recovered on plate medium at 28°C. Then the single colony was cultured in a tryptic soy broth soybean-casein digest medium (pancreatic digest of casein 17.0 g L^−1^, papaic digest of soybean 3.0 g L^−1^, dextrose 2.5 g L^−1^, sodium chloride 5.0 g L^−1^, dipotassium phosphate 2.5 g L^−1^) in a shaker incubator at 180 rpm at 28°C. The colony forming units per milliliter (CFU/mL) in the final culture were measured by plate counting under a microscope. Bacterial suspensions at 1.0 × 10^7^ CFU/mL were prepared in normal saline.

For the challenge experiment, largemouth bass in all groups were firstly anaesthetized with MS 222 and then artificially injected with the *N. seriolae* (0.10 mL) by intraperitoneal injection. After challenge experiment, all fish was transferred back to each tank with the same water conditions. Fish samples were obtained from FM and CPC group at 0 day, 1 day, and 3 days post infection (dpi). Fish sampling methods were similar to previous in 2.3.

### Histological assay

The liver and gill tissues of largemouth bass in two groups at different time points post infection were fixed with polyformaldehyde. After fixing, the tissues were transfered into gradient alcohol for dehydration, xylene for transparent, and then paraffin for section slicing. The obtained 5-μm sections of liver tissues were then used for H.&E. staining, following the producers’ introduction. The stained slides were examined under a light microscope (Olympus, DP72) equipped with a camera (Nikon E600) and CellSens Standard Software (Olympus) for image acquisition.

### TUNEL assay

Terminal deoxynucleotidyl transferase dUTP nick-end labeling (TUNEL) assay was conducted to precisely detect cells experiencing programmed cell death (PCD) with the one-step TUNEL Assay kit (Beyotime, China) following the manufacturer’s instructions. Briefly, tissue sections were twice dewaxed in xylene for 5-10 minutes. Then tissues were immersed into anhydrous ethanol for 5 minutes, 90% ethanol for 2 minutes, 70% ethanol for 2 minutes, and distilled water for 2 minutes. Tissues were treated with DNase-free proteinase K (Beyotime, 20μg/mL) at room temperature for 15-30 minutes and then washed with PBS for three times to remove excess proteinase K. Afterwards, the labelling solution containing terminal deoxynucleotidyl transferase, buffer, and fluorescein dUTP was added to tissues for labelling reaction at 37 °C for 60 minutes in a humidity chamber. Following incubation, excess labelling solution was washed off smears with PBS, and then cell smears were mounted with fluorescent microscopy mounting solution. Images were captured and analyzed using a CCD camera (Olympus, Japan).

### RNA extraction and RT-qPCR analysis

Total RNA samples were extracted from liver and gill tissues using TRIZOL reagent (Invitrogen, Carlsbad, CA, USA) according to the manufacturer’s instruction. The purity and concentration of RNA were detected with Nanodrop 2000 spectrophotometer (Thermo scientific, USA), accompanied with the integrity of RNA checked by 1.0% agarose gel electrophoresis. One microgram of the resulting total RNA was reverse transcribed into cDNA using the SuperScript III RNaseH-Reverse Transcriptase kit (Invitrogen, Carlsbad, CA, USA) and oligo dT primers (Promega, Charbonnie `res, France) according to the manufacturers’ instructions.

Quantitative RT-PCR was carried out on an iCycler iQTM real-time PCR detection system (BIO-RAD, Hercules, CA, USA) using the Eva Green 2 × qPCR Master mix (ABM, Canada). The program was as follows: 95 °C for 5min, followed by 40 cycles of 95 °C for 10 s and 60 °C for 30 s. In addition, a melt curve analysis was performed after amplification to verify the accuracy of each amplicon. 18S was employed as a non-regulated reference gene and no changes in 18S gene expression were observed in our investigations (data not shown). The relative quantification of the target genes was determined *via* the comparative CT method (2^-ΔΔCt^ method). Primer used in the present study was shown in **Table 2**.

**Table 2:** Primers used in the present study.

Gene Name	Abbreviation	Gene ID	Forward Sequence	Reverse Sequence
18S ribosomal RNA	*18s*	119916718	GCAAAGCTGAAACTTAAAGGAATTG	TCCCGTGTTGAGTCAAATTAAGC
myocyte enhancer factor 2a	*mef2a*	119893304	GCAGTTTGGGGAAGGTCATA	GAGCTGATGCCGGAGTAGAC
myocyte enhancer factor 2b	*mef2b*	119900933	GCACCAACACGGACATACTG	TTCTCACAGCCTGCAGACAC
myogenin	*myog*	119886574	AGTTGGGGTGACAGGAACAG	GCCTCGTTCACCTTCTTCAG
myogenic differentiation 1	*myod1*	119894171	TGCCGCTGATGATTTCTATG	GAGGAGGAAGAGGAGGAGGA
myostatin a	*mstn*	119900535	TTTAGCGTTCAGCAATGACG	GCCACGCAAACCAATTTACT
NACHT, LRR and PYD domains-containing protein 3-like	*nlrp3*	119896601	TCAATCACGCAAAGTATGG	CTCAAGTGGGCAAAGCAG
NLR family, CARD domain containing 3	*nlrc3*	119916443	TGTACTGCAGCCATCTCAGG	AGAGCCGATGTTGTTGTTCC
caspase3	*casp3*	119888653	GCTTCATTCGTCTGTGTTC	CGAAAAAGTGATGTGAGGTA
caspase-1-like	*casp1*	119883616	ATAGCAGCAGAGCAGACA	AGGACAGACCCAGTTCAT
apoptosis-associated speck-like protein containing a CARD	*asc*	119897170	AAAGGCAATAGCAGACGC	AAGTGGAAACCAGGATGT
gasdermin-E-like	*gsdme*	119897735	TTACTTGGTATCTGGTGGTG	ACATTCATCTGTCGTGGC
interleukin 1, beta	*il-1β*	119914255	ACATGACGGAAGTTCAGGAT	GCTGCCTGCTATAGTTGGTT
interleukin-8	*il-8*	119892024	CGTTGAACAGACTGGGAGAGATG	AGTGGGATGGCTTCATTATCTTGT
interleukin-18-like	*il-18*	119882624	CCCTGTTGCTTATCCTGT	CACCATCTTCTTGCCATC
hepcidin antimicrobial peptide	*hep*	119897237	ACACTCGTGCTCGCCTTTAT	AGAAGCCACAGCCCTTGTTA
serum amyloid A	*saa*	119890486	GTTGAAGCTGCGAAAGGAAC	AGCCACTCTCTGGCATCACT
immunoglobulin T	*igt*		GAAGGTCAACAACGCTGAGTG	TGTTGCTGGTCACATCTAGTCC
immunoglobulin D	*igd*		AAGGGAAACAGTGCTGTGCT	TTGCCAGTGGGGTTTGACTT
immunoglobulin M	*igm*	119887406	CTCAATGACCCCCCCTAA	CAAGCCAAGACACCAAAA
T cell receptor alpha	*tcrα*	119903280	CATGAACCACTGGCTGAGAA	TCAGCAGCAATGACATCTCC
T-cell receptor beta-1	*tcrβ*	119917160	CGGACCATGTGAGTGTGTTC	TGAAGAAGTTGACGGTGCAG
cd8 beta	*cd8*	119885986	CGCAGTATCTCCAGCCAGAT	CCAGGCTTCCACATTTCTGT
LCK proto-oncogene Src family tyrosine kinase	*lck*	119910103	TACAGAATCCGCAACATGGA	CTCCAGCTTCAGGGACTCAC

### Western blotting analysis

Proteins from liver homogenates were separated on SDS-PAGE. For each assay, all samples were analyzed at the same time on four-wide gels to eliminate inter-assay variation. Proteins were electrophoretically transferred to polyvinylidene difluoride transfer membranes (Pall Corporation), which were incubated with appropriate primary antibodies, washed and exposed to an appropriate secondary antibody. Blots were developed using an enhanced chemiluminescence kit (Beyotime Institute of Biotechnology) and visualized. Primary antibodies for Phospho-S6 (Ser235/236) (cat # 4856) and S6 (cat # 2217) used in the present study were purchased from Cell Signaling Technology (U.S.A.) and primary antibody for β-actin (AC004) was purchased from ABlonal Biotechnology (China).

### Immunofluorescent assay

In order to detect T cells, liver paraffin sections were stained with Alexa Fluor 647 anti-Lck (pAb, 1: 900) overnight at 4°C. After washing four times, all sections were stained with DAPI (4′,6-diamidino-2-phenylindole, 1 μg/mL, Invitrogen) before mounting. Images were captured and analyzed using a CCD camera (Olympus, Japan).

### Calculations and statistical analysis

Mean final weight (MFW, g) = total final weight/fish number; Weight gain ratio (WGR, %) = 100*(final body weight – initial body weight)/initial body weight; Specific growth rate (SGR, %/day) = 100* (ln final body weight − ln initial body weight)/days; Feed intake ratio (FI, %/day) = 100 *dry feed intake/[(initial body weight + final body weight)/2]/days; Feed conversion ratio (FCR) = dry feed intake/(final body weight – initial body weight).

All statistical analyses were performed using SPSS 17.0. Data about fish growth performance, feed utilization and western blotting were analyzed using student’s t-test. All gene-expression results were tested for normality using the Shapiro Wilk’s-W’s test, and normally distributed data were analyzed by Factorial (two-way) analysis of variance (ANOVA) to determine the main effects of period and group, and their interactions on gene expression. When significant interaction of period and group were observed, data were analyzed by one-way ANOVA followed by Tukey’s multiple range tests to inspect differences among all the treatments. When only a significant interaction and significant main effects of period or group were observed, data were analyzed by one-way ANOVA followed by Tukey’s multiple range tests to inspect differences among periods within each group and vice versa. When the significance is only with the main effects of period or group, the data were analyzed by the two-way ANOVA followed by Tukey’s multiple range tests to assess the main effects of period or group only. Differences were considered significant when *P* < 0.05. All data were expressed as mean ± standard deviation of the mean (SD), except the specific statement.

## Results

### Largemouth bass growth performance and feed utilization were not affected after CPC replacing dietary fishmeal for 8 weeks

The growth performance of juvenile largemouth bass after 8-weeks feeding trial in the present study is shown in **Table 3**. Mean final weight (MFW) of juvenile largemouth bass in two groups could reach 95.42 ± 0.64 and 94.78 ± 0.90, respectively, which showed no significant difference between two groups (*P*>0.05). Accordingly, the weight gain ratio (WGR) and specific growth ratio (SGR) of bass were also around 1.96 and 1.80 in two groups, respectively. Moreover, the feed intake ratio (FI) also reached 0.23 ± 0.03 and 0.28 ± 0.06, which difference was also not significant (*P*>0.05). Feed conversion ratio was 0.89 ± 0.13 and 1.06 ± 0.03 for FM and CPC, which were not significantly different (*P*>0.05). Thus, the growth performance and feed utilization of largemouth bass was not significantly affected after CPC replacing dietary fishmeal for an 8-weeks feeding period.

**Table 3:** Growth parameters of juvenile largemouth bass in the present study.

	Control	CPC	P value
Mean initial weight (MFW, g)	32.2 ± 0.12	32.1 ± 0.04	0.350
Mean final weight (MFW, g)	95.42 ± 0.64	94.78 ± 0.90	0.461
Specific growth ratio(SGR,%/day)	1.81 ± 0.005	1.80 ± 0.01	0.581
Weight gain ratio (WGR, %)	1.96 ± 0.009	1.95 ± 0.02	0.584
Feed intake ratio (FI, %/day)	1.58 ± 0.22	1.88 ± 0.04	0.137
Feed conversion ratio (FCR)	0.89 ± 0.13	1.06 ± 0.03	0.138

### Largemouth bass exhibited better growth potential after adaptation to dietary CPC

**Figure 1:**
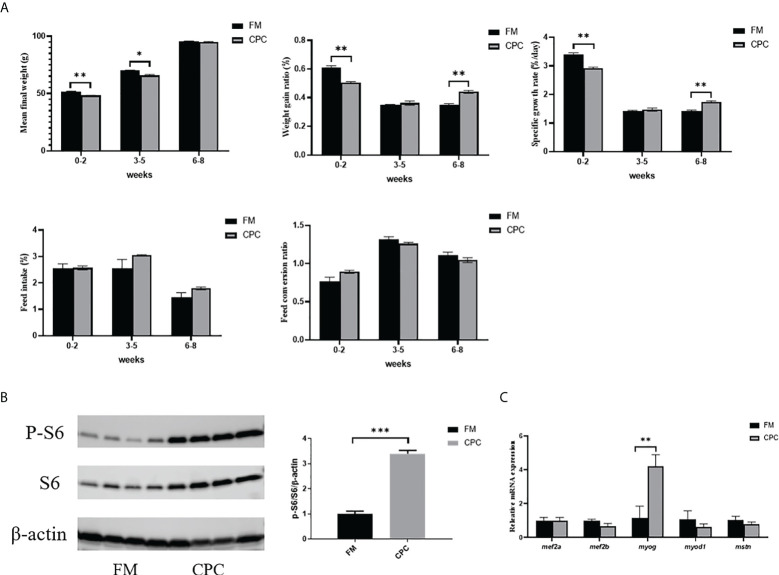
Largemouth bass exhibited better growth potential after adaptation to dietary CPC. **(A)** The growth parameters including mean final weight (MFW, g), weight gain ratio (WGR, %), specific growth ratio (SGR, %/day), feed intake ratio (FI, %/day) and feed conversion ratio (FCR) of juvenile largemouth bass fed with FM and CPC in 0-2 weeks, 3-5weeks and 6-8 weeks. **(B)** The relative protein level of S6 and P-S6 in liver of juvenile largemouth bass fed with FM and CPC. **(C)** The relative mRNA expression levels of genes involved in muscle formation (*mef2a, mef2b, myog, myod1, mstn*) in the liver of juvenile largemouth bass in both FM group and CPC group. All data was analyzed with student’s t-test and data are means ± SD (n = 6). *P< 0.05; **P< 0.01; ***P< 0.001.

In order to have a more systematic overlook of bass growth performance with two diets, the growth parameters were separately evaluated from 0-2 weeks, 3-5 weeks and 6-8 weeks (**Figure 1A**). In the first two weeks, juvenile largemouth bass in FM group exhibited better MFW, WGR and SGR than bass in CPC group. However, there were no significant differences in WGR and SGR between two groups (*P*>0.05) during the period from 3^rd^ weeks to 5^th^ weeks. Furthermore, both WGR and SGR of bass in CPC group was even significantly higher than that in FM group (*P*<0.05) in the last 3 weeks, and MFW in two groups showed no significant difference (*P*>0.05). Thus, largemouth bass exhibited adaptation to dietary CPC and then showed better growth potential in the prolonged rearing period.

In order to confirm the growth potential at 8 weeks after adaptation to dietary CPC, the activation status of TOR signaling pathway was evaluated *via* western blotting analysis. As shown in **Figure 1B**, the phosphorylation level of S6 was significantly higher in bass liver of CPC group than that of FM group (*P*<0.05). Additionally, the relative mRNA expression level of genes involved in myogenesis were also examined by RT-qPCR. As shown in **Figure 1C**, the expression of myocyte enhancer factor 2a (*mef2a*), myocyte enhancer factor 2b (*mef2b*), myostatin a (*mstn*), and myogenic differentiation 1 (*myod1*) showed no significant difference between two groups (*P*>0.05), while the expression of myogenin (*myog*) in bass liver of CPC group was also significantly higher than that of FM group (*P*<0.05).

### Largemouth bass liver structure along with the programmed cell death was not significantly affected after dietary CPC replacing fishmeal

**Figure 2:**
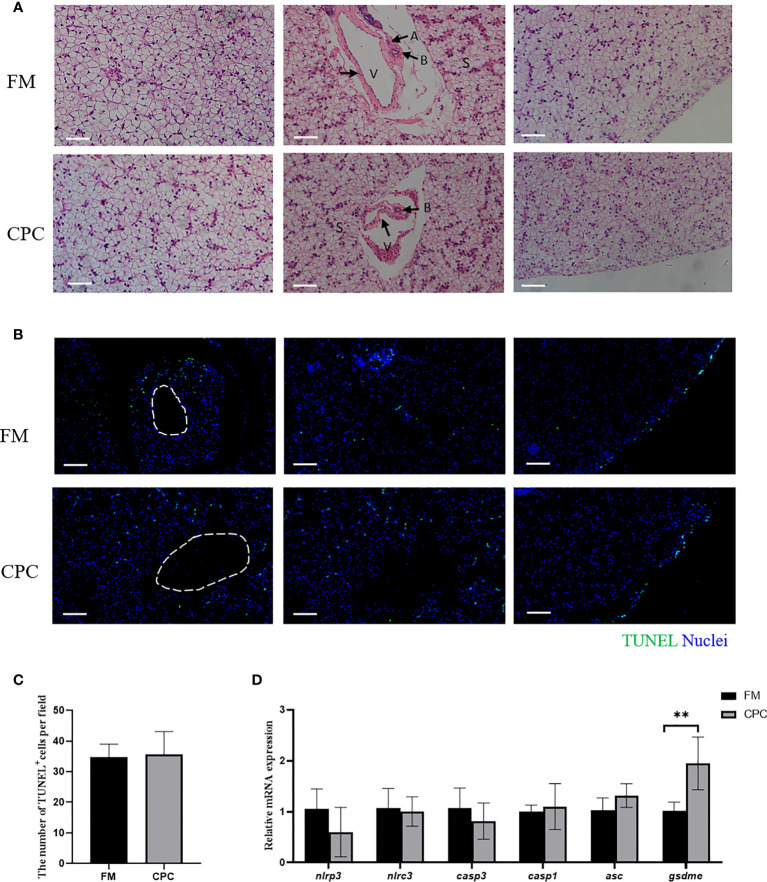
Largemouth bass liver structure along with the programmed cell death was not significantly affected after dietary CPC replacing fishmeal. **(A)** The H.E. staining results of juvenile largemouth bass liver fed with FM and CPC for 8 weeks. V: veins; B: bile ducts; A: arterial; S: sinusoid. Scale bars, 50μm. **(B)** TUNEL assay with immunofluorescence microscope on liver tissue to detect the cells experiencing programmed cell death. Scale bars, 100μm. **(C)** The calculated number of TUNEL^+^ cells in liver of two groups. **(D)** The relative mRNA expression levels of genes involved in apoptosis and pyroptosis (*nlrp3, nlrc3*, *casp1, casp3, asc* and *gsdme*) in the liver of juvenile largemouth bass in both FM group and CPC group. All data was analyzed with student’s t-test and data are means ± SD (n = 6). *P< 0.05; **P< 0.01; ***P< 0.001.

The liver structure of bass in two groups was evaluated *via* H.&E. staining (**Figure 2A**). As shown, the bass liver exhibited cuboidal hepatocytes with rounded nuclei which are arranged in cords and supported by reticulated fibers and connective tissue. The sinusoidal gaps between the cords are also known as hepatic blood sinuses (S), which are the sites of blood filtration into the liver. Moreover, veins (V) and bile ducts (B) are also detected to be accompanied with each other while arterial (A) features were rarely observed. No significant differences were detected in liver interior (left), hepatic blood sinuses (middle) and edge (right) of juvenile largemouth bass in FM and CPC group.

Moreover, the programmed cell death (PCD) including apoptosis and pyroptosis in bass liver was also detected by TUNEL. As shown in **Figure 2B**, TUNEL^+^ cells could be mainly detected at the edge of the liver tissue in juvenile largemouth bass of two groups. Additionally, few cells experiencing PCD could be detected in the interior of liver tissue. Statistical analysis showed that the number of TUNEL^+^ cells in two groups were not significantly different (*P*>0.05) (**Figure 2C**). Additionally, the mRNA expression levels of genes involved in apoptosis and pyroptosis were also evaluated in bass liver of two groups. As shown in **Figure 2D**, the expression of *NACHT, LRR and* PYD domains-containing protein 3-like (*nlrp3*), NLR family CARD domain containing 3 (*nlrc3*), caspase-1-like (*casp1*), caspase-3-like (*casp3*) and apoptosis-associated speck-like protein containing a CARD (*asc*) showed no significant difference in two groups (*P*>0.05). However, the expression of gasdermin-E-like (*gsdme)* in CPC group was significantly higher than that in FM group (*P*<0.05).

### Largemouth bass hepatic immunity was not significantly affected after CPC replacing fishmeal

**Figure 3:**
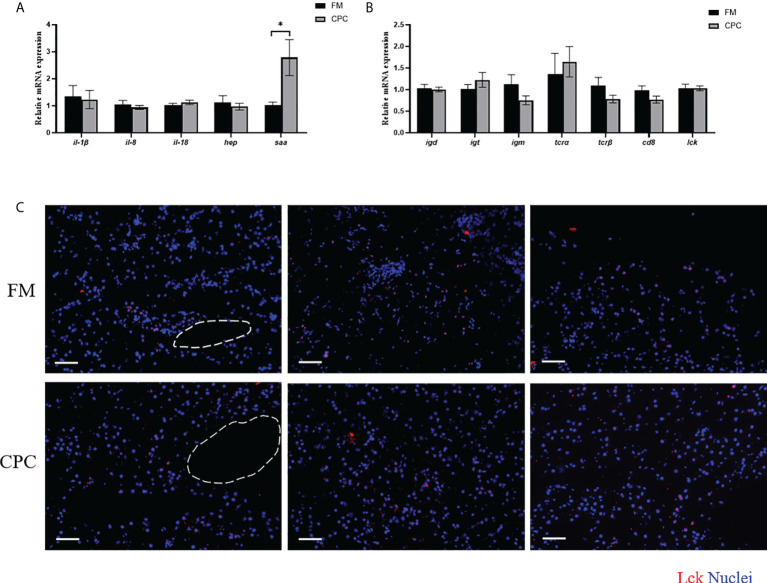
Largemouth bass hepatic immunity was not significantly affected after CPC replacing fishmeal. **(A)** The relative mRNA expression levels of genes involved in innate immunity (*il-1β, il-18, il-8*, *hep* and *saa*) in the liver of juvenile largemouth bass in both FM group and CPC group. **(B)** The relative mRNA expression levels of genes involved in adaptive immunity (*igm, igd, igt*, *tcrα, tcrβ, lck*, and *cd8)* in the liver of juvenile largemouth bass in both FM group and CPC group. **(C)** Immunofluorence results of T cells in liver of juvenile largemouth bass in both FM group and CPC group with antibody against Lck. Scale bars, 100μm. All data was analyzed with student’s t-test and data are means ± SD (n = 6). *P< 0.05; **P< 0.01; ***P< 0.001.

The expression of genes involved in the innate immunity of largemouth bass liver in two groups were evaluated by RT-qPCR. As shown in **Figure 3A**, the expression of interleukins including interleukin-1beta (*il-1β*), interleukin-18-like (*il-18*), and interleukin-8 (*il-8*) *s*howed no significant differencein bass liver of two groups (*P*>0.05). Similarly, the expression of hepcidin antimicrobial peptide (*hep*) was also not significantly affected by dietary CPC inclusion (*P*>0.05). However, dietary CPC inclusion significantly increased the expression of serum amyloid A (*saa*) (*P*<0.05).

Meanwhile, the adaptive immunity in bass liver was also evaluated *via* both detecting the expression of related genes and the relative abundance of T cells. As shown in **Figure 3B**, the expression of immunoglobulins including *immunoglobulin M* (*igm*)*, igd*, and *igt* showed no significant difference in largemouth bass liver of two groups (*P*>0.05). Similarly, the expression levels of T cell receptor alpha (*tcr*α), T-cell receptor beta-1 (*tcrβ*), LCK proto-oncogene Src family tyrosine kinase (*lck*), and cd8 beta (*cd8*) were also not significantly (*P*>0.05) affected by dietary CPC inclusion. Moreover, the relative abundance of T cells in bass liver were also determined using immunofluorescent assay with antibody against Lck, a cytoplasmic protein bound to CD4 and CD8 in T lymphocytes. As shown in **Figure 3C**, Lck^+^ cells could be detected in bass liver tissue of two groups, which are diffusely distributed in the hepatic sinusoids. The number of Lck^+^ B cells in bass liver showed no significant difference between two groups (*P*>0.05).

### Higher inflammatory responses were induced in both liver and gill of largemouth bass fed with CPC after *N. seriolae* infection

**Figure 4:**
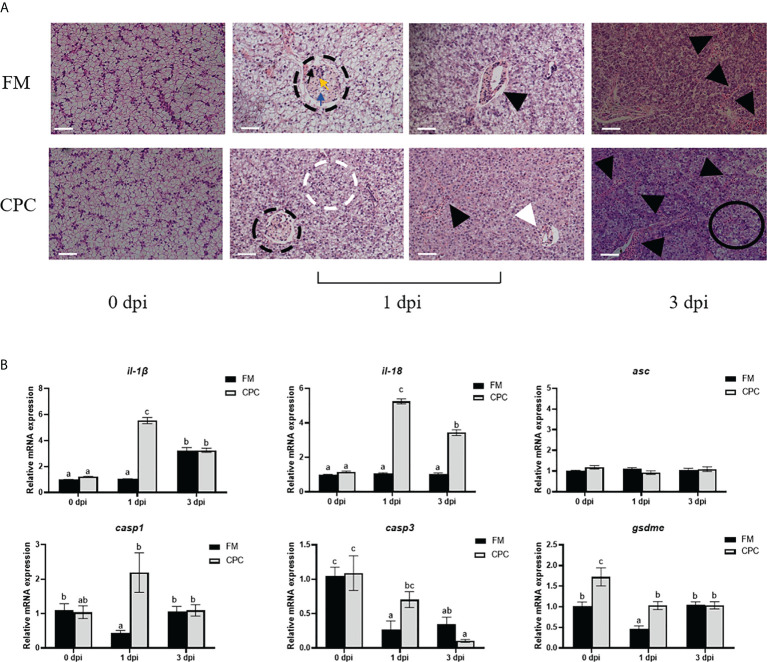
Higher inflammatory responses were induced in liver of largemouth bass fed with CPC after *N. seriolae* infection. **(A)** The H.E. staining results of juvenile largemouth bass liver fed with FM and CPC at different days post *N. seriolae* infection (dpi). Blue arrows: lipid droplets; black arrows: macrophages; yellow arrows: lymphocytes; black dotted circles: lipogranulomas; white dotted circles: ruptured cell membranes; white triangles: lymphocytic infiltration; black triangles: inflammatory cells; black circles:cell vacuolar degeneration. Scale bars, 50μm. **(B)** The relative mRNA expression levels of genes involved in inflammation and pyroptosis (*il-1β, il-18, asc, casp1, casp3* and *gsdme*) in the liver of largemouth bass fed with FM and CPC at different days post *N. seriolae* infection (dpi). All data was analyzed with two-way ANOVA and data are means ± SD (n = 6). Mean values with different letters indicated significant difference among groups, P < 0.05.

*N. seriolae* infection induced serious pathological changes in liver of largemouth bass, which were detected *via* H.&E. staining. As shown in **Figure 4A**, lipogranulomas (black dotted circles) composed of a mixture of lipid droplets (blue arrows), macrophages (black arrows), and lymphocytes (yellow arrows) could be detected in liver of FM and CPC group at 1 dpi. Meanwhile, the number of inflammatory cells (black triangles) in liver of CPC group were more than that in FM group, accompanied with the irregularly arranged cells and ruptured membranes (white dotted circles) in some area. At 3 dpi, the pathological changes were more serious, with chaotic arrangement of hepatocytes and aggravated inflammatory response. Accordingly, a large number of eosinophils infiltrating in liver tissue could be detected in CPC group, which is more serious than that in FM group. Severe cellular vacuolar degeneration (black circles) could also be detected in the CPC group. Moreover, the expression of genes involved in inflammatory responses and pyroptosis in liver were also evaluated *via* RT-qPCR. As shown in **Figure 4B**, *N. seriolae* infection induced the upregulated expression of *il-1β* in FM group at 3 dpi, while CPC group induced earlier upregulation of *il-1β* expression at 1 dpi (*P*<0.05). Moreover, *N. seriolae* infection induced the upregulated expression of *il-18* in liver of CPC group at 1 dpi and 3 dpi, whose expression was significantly higher than that of FM group (*P*<0.05). Accordingly, the expression of *casp1* and *casp3* at 1 dpi was also significantly higher in liver of CPC group than that of FM group. Similarly, the expression of *gsdme* at both 0 dpi and 1 dpi was also higher in liver of CPC group than that of FM group (*P*<0.05). Thus, dietary CPC inclusion may trigger high inflammatory potential in bass liver during *N. seriolae* infection, which is in accordance with the histological structure.

**Figure 5:**
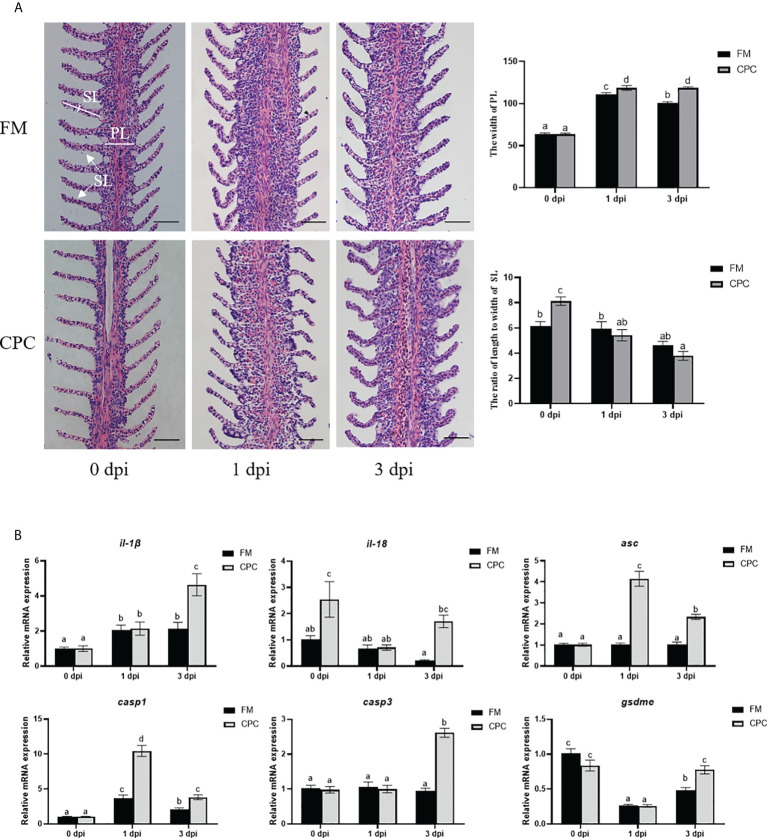
Higher inflammatory responses were induced in gill of largemouth bass fed with CPC after *N. seriolae* infection. **(A)** The H.E. staining results of juvenile largemouth bass gill fed with FM and CPC at different days post *N. seriolae* infection (dpi). PL: primary gill lamella; SL: secondary gill lamella. Scale bars, 50μm. **(B)** The relative mRNA expression levels of genes involved in inflammation and pyroptosis (*il-1β, il-18, asc, casp1, casp3* and *gsdme*) in the gill of largemouth bass fed with FM and CPC at different days post *N. seriolae* infection (dpi). All data was analyzed with two-way ANOVA and data are means ± SD (n = 6). Mean values with different letters indicated significant difference among groups, P < 0.05.

In order to further confirm the pro-inflammatory effects of CPC during bacterial infection, the histological structures and expression of genes involved in inflammatory responses of gill were also evaluated (**Figure 5**). As shown in **Figure 5A**, *N. seriolae* infection induced serious histological structural changes in gill, as the primary gill lamella (PL) thickened, which could be detected in two groups. Statistical analysis indicated that the width of PL was significantly higher in CPC group than that in FM group at 1dpi and 3 dpi (*P*<0.05). Meanwhile, the secondary gill lamella (SL) turned to be short and bending after infection in both groups. The ratio of length to width of SL in CPC group was significantly higher than that in FM group before infection, but turned to be much lower in CPC group at both 1 dpi and 3 dpi (*P*<0.05). Moreover, *N. seriolae* infection also induced the upregulated expression of *il-1β* and *casp1* in the gill of largemouth bass in FM group (**Figure 5B**). The expression of *il-18* and *il-1β* in the gill of CPC group was even significantly higher than that of FM group at 3 dpi, while CPC group also exhibited higher expression of *il-18* at 0 dpi (*P*<0.05). The expression of *casp1* and *asc* in the gill were also significantly higher in CPC group than FM group at both 1 dpi and 3 dpi. Accordingly, the expression of *casp3* and *gsdme* in the gill were also significantly higher in CPC group than FM group at 3 dpi (*P*<0.05).

## Discussion

Cottonseed is a sustainable source of plant protein, producing ~10 million metric tons of protein globally (22). In aquaculture practice, cottonseed meal has been widely tested in many fish species, while the existing gossypol seriously restricted its application (23). Reducing the gossypol content to permissible limits (450 ppm, World Health Organization) *via* physical, chemical and biological methods makes cottonseed a good candidate for use in food and feed formulations, with a balanced amino acid composition and functional properties comparable to those of soy protein (24). Recently, the new product, CPC, has been proved to effectively replace partial FM without adverse effects on growth of several aquatic animal species (10, 25). Our present study illustrated the dietary inclusion of CPC (15%) in largemouth bass diet without affecting fish growth performance when fishmeal decreased from 48% to 35% (**Table 3**). Early studies have also tested the effects of CPC replacing fishmeal in largemouth diet, while the replacing level varied in different studies (18–20). Considering the basic fishmeal level and other protein sources varied in different studies, it will be more convincible to use the real inclusion level of CPC in fish diet. For example, He et al. (18) reported that CPC inclusion of up to 13% showed no adverse effects on weight gain, specific growth rate and protein efficiency ratio of largemouth bass, while Xie et al. (19) also reported that CPC inclusion (23.5%) even promoted largemouth bass growth performance. Another study reported that CPC inclusion level (no more than 36.23%) could replace fishmeal in bass diet (initial body weight of 95.3g) without compromising the growth, flesh composition, texture, flavor characteristics and antioxidant capacity (20). Such phenomenon is rather common in many studies of fishmeal replacement by other protein sources. For example, in hybrid grouper (*Mycteroperca tigris × Epinephelus lanceolatus*), dietary CPC could replace fishmeal of up to 60% without significantly altering the growth performance but adversely influenced fish intestinal immune responses and microbial profiles (26), while the same research group showed that dietary CPC inclusion at low levels (15.76% and 18.18%) even promote hybrid grouper growth performance (27). One important question remains why results varied in the different reports. It has been speculated that differential results may originate from the varied fish growth period, the different basic diet formula, and different rearing environment. Here, in order to explain the question, the growth parameters in different feeding period were systemically evaluated. As shown in **Figure 1A**, bass in CPC group showed lower WGR and SGR than that in FM group in the first two weeks, while no difference was detected between two groups in the next 3weeks. However, both WGR and SGR of bass in CPC group was even significantly higher than that in FM group in the last 3 weeks (**Figure 1A**), which suggested that largemouth bass may have adapted to CPC diet and exhibited better growth potential at the end of 8 weeks feeding trial. A recent study reported that largemouth bass even showed adaptation of dietary glucose after 8 weeks feeding trial (28). Former studies in rainbow trout have also showed that rainbow trout can be adapted and even trained to plant-sourced proteins and exhibited good performance after several generations of selection (29), which all supported our hypothesis. Additionally, our previous studies have proved that TOR functions as the central regulator of fish growth and nutrient metabolism, and the activation status could be selected as the marker of fish growth potential (30). For example, the inhibition of TOR by rapamycin significantly resulted in the decreased fish body weight, while the adverse growth performance during fishmeal replacement by plant proteins could also be attributed to the inhibition of TOR signaling. In order to further confirm the previous assumption that largemouth bass showed better growth potential after 8-weeks adaptation to dietary CPC, the activation status of TOR signaling pathway was evaluated. In accordance with the higher SGR and WGR from 6-8 weeks, the phosphorylation level of S6 was significantly higher in bass liver of CPC group than that of FM group (**Figure 1B**). Moreover, the RNA expression level of *myog*, which plays a key role in muscle differentiation by controlling myoblast fusion and myofiber formation, was also significantly higher in bass liver of CPC group than that of FM group (**Figure 1C**). All these results may suggest that largemouth bass has adapted to dietary CPC and then exhibited even better growth potential with prolonged feeding period. Such results may also explain that fish fed with plant proteins in some studies may exhibit better growth performance than that fed with fishmeal (27), which have adapted to such plant protein sources.

Besides the growth performance, fish health has received attention in recent years, especially during the fishmeal replacement by plant proteins. In the aquaculture practice of largemouth bass, liver health has been given special attention from fish farmers and scientists, which needs a comprehensive overlook of liver structures and functions. As known in mammals, liver is an essential organ controlling nutrient metabolism and immune responses, which is closely related to its unique pattern of vascular distribution, namely, the venous-biliary-artery tract (VBAT) structure (31). Such mammalian-like VBAT structures also exist in some teleost species, such as Atlantic salmon (*Salmo salar*), rainbow trout (*Oncorhynchus mykiss*) and brown trout (*Salmo trutta*) (32). Here in the present study, the typical VBAT structure was also detected in bass liver *via* H.&E. staining. As shown in **Figure 2A** bass liver, cuboidal hepatocytes with rounded nuclei were arranged in cords and supported by reticulated fibers and connective tissue. Moreover, veins (V) and bile ducts (B) are also detected to be accompanied with each other while arterial (A) features were rarely observed. Moreover, no significant difference was found in liver structure of juvenile largemouth bass between two groups. The tissue homeostasis could be due to the controlled proliferation and programmed cell death (PCD) of inner cells (33). Here, TUNEL assay was conducted to evaluate the programmed cell death (**Figure 2B**). Results also showed that TUNEL^+^ cells were mainly distributed at the edge of liver with few apoptotic cells in the interior of bass liver and no significant difference was detected between FM and CPC group (**Figure 2C**). Moreover, the expression levels of several PCD-related genes were also evaluated. As shown in **Figure 2C**, excepting *gsdme*, the expression of genes including *nlrp3, nlrc3*, *casp1, casp3* and *asc* showed no significant difference between two groups. Thus, H.&E. staining, TUNEL assay and RT-qPCR results all suggested that bass liver structures were not significantly affected after dietary CPC inclusion at 15% for 8 weeks.

As mentioned above, fish liver is also involved in the immune responses, as several types of immune cells have been identified in the hepatic blood sinuses (34). Dietary and commensal bacterial products from the gastrointestinal tract enters liver from portal vein, which exert liver to continuous inflammatory potential (35). In fact, fish is the first bony vertebrate to develop both innate and adaptive immunity among all vertebrates (36), thus both innate and adaptive immune responses in liver were also systemically evaluated. Inflammatory responses are soon induced after pathogenic infection or other damage signals exposure. The expression levels of genes involved in the inflammatory responses including *il-1β, il-8* and *il-18* showed no significant difference between two groups. Moreover, the expression levels of several antibacterial peptide including *hep* and *saa* were also evaluated. The expression level of *saa*, which plays a key role in bacterial clearance and inflammatory regulation, in CPC group was significantly higher than that in FM group, which suggested that bass liver in CPC group was activated to control the inflammatory responses. On the other hand, the adaptive immunity in fish is executed by B cells and its secreted immunoglobulins which are responsible for humoral immunity, and T cells which are responsible for cellular immunity (37). As reported, three types of immunoglobulins (Igs) have been identified in teleosts (36). In the present study, the expression of Igs including *igm, igt* and *igd*, also showed no significant difference between two groups. Moreover, T cells are characterized by the presence of T-cell receptors (TCRs), which consists of an α (TCRα) chain and a β (TCRβ) chain in the conventional T cells. Such T lymphocoytes could be distinguished by the expression of mutually exclusive CD4^+^ or CD8^+^ co-receptors (38). Additionally, Lck is a cytoplasmic protein bound to CD4 and CD8 in T cells, which participates in antigen-induced T cell activation (39). Here, in the present study, the expression of *tcra, tcr*β*, lck*, and *cd8* also showed no significant difference between two groups, which indicated that dietary CPC inclusion did not significantly affect the expression of T cell markers. Additionally, immunofluorescence study with antibody against Lck also showed that T cells also exist in the liver interior with no Lck^+^ cells detected in the blood vessel, which indicated the successful perfusion procedure. Importantly, the number of T cells showed no significant difference between two groups. Thus, the innate and adaptive immunity in bass liver under normal status were not significantly affected during dietary CPC inclusion.

Largemouth bass is rather sensitive to *N. seriolae* infection, which caused serious hepatic damage and even mortality (14). In order to further illustrate the immune responses of bass liver during pathogenic infection, all bass was injected with *N.a seriolae*. As shown in **Figure 4**, the liver pathological structures showed rather serious damages. Lipogranulomas could be detected in liver of FM and CPC group at 1 dpi, while severe cellular vacuolar degeneration (black circles) could also be detected in the CPC group at 3 dpi. Moreover, the number of inflammatory cells (black triangles) in liver increased with the prolonged infection period, and such inflammatory cells in liver of CPC group were more than that in FM group, accompanied with the irregularly arranged cells and ruptured membranes. As mentioned above, fish liver is continuously exposed to dietary and commensal bacterial products, which results in persistent, regulated inflammation (16). When fishmeal was replaced by plant proteins in fish diet, the plant sources-originated anti-nutritional factors would cause further continual inflammatory stimulus to liver. Failure to clear such ‘dangerous’ stimuli may lead to chronic pathological inflammation and even disrupted tissue homeostasis such as fibrosis, cirrhosis and eventual liver failure (17). Recently, one new type of PCD, pyroptosis which is executed by gasdermin family proteins, has been discovered to closely correlate with the inflammation (40). Under the stimulation of pathogen- and/or damage-associated molecular patterns, pattern recognition receptors (PRRs) such as Nod like receptors could recruit apoptosis-associated speck-like protein containing a CARD (ASC) and pro-caspases to form inflammasomes and then activate caspases, which then cleave gasdermin E in fish to form oligomeric pores, and also promote the maturation and release of inflammatory cytokines such as *il-1β* and *il-18* (40). In the present study, *N. seriolae* infection induced the upregulated expression of *il-1β* at 3 dpi in the FM group, while *il-1β* expression in CPC group was significantly induced much earlier at 1 dpi. Moreover, *N. seriolae* infection induced the upregulated expression of *il-18* in the liver of CPC group at both 1 dpi and 3 dpi, whose expression was significantly higher than that of FM group. Besides the inflammatory cytokines, the expression of *casp1* and *casp3* was also significantly higher in bass liver of CPC group than that of FM group at 1 dpi. Similar results were detected in the expression of *gsdme* in liver at 0 dpi and 1 dpi. Thus, dietary CPC inclusion may trigger high inflammatory potential after *N. seriolae* infection, which is in accordance with the histological structure.

In order to further confirm the pro-inflammatory effects of CPC during bacterial infection, the histological structures and expression of genes in gill involved in inflammatory responses were also evaluated. As shown in **Figure 5A**, *N. seriolae* infection induced serious histological structural changes in gill, as the primary gill lamella (PL) thickened and the secondary gill lamella (SL) shortened, which could be detected in two groups. Earlier studies in hybrid snakehead (*Channa maculata* ♀ × *Channa argus* ♂) also detected that the gill filament was become short, twisted and shrunk seriously after *N. seriolae* infection (41), with a number of pale yellow nodules could be detected on the serosal surface, mesentery and internal organs (gill, heart, spleen, swim bladder, kidney and liver). Especially, more serious gill damages could be detected in CPC group, with much higher the width of PL but lower ratio of length to width of SL than that in FM group. Moreover, *N. seriolae* infection also induced the upregulated expression of *il-1β*, and *casp1* in the gill of largemouth bass (**Figure 5B**). Moreover, the expression of *il-18* and *il-1β* in the gill of CPC group was even significantly higher than that of FM group at 3dpi, while CPC group also exhibited higher expression of *il-18* at 0 dpi. The expression of *casp1* and *asc* in the gill were also significantly higher in CPC group than FM group at both 1 dpi and 3 dpi. Accordingly, the expression of *casp3* and *gsdme* in the gill were also significantly higher in CPC group than FM group at 3 dpi. All these results in gill further confirmed that dietary CPC inclusion in largemouth bass significantly increased inflammatory potential and pyroptosis during bacterial infection, which may further influence fish health and survival during infection.

In summary, our present study indicated the potential of dietary CPC as replacement sources for fish meal, and that largemouth bass can adapt to it and even showed better growth potential. However, special concern should be given during the application of CPC in largemouth bass diet as it may induce higher inflammatory risk during *N. seriolae* infection.

## Data availability statement

The original contributions presented in the study are included in the article/supplementary material. Further inquiries can be directed to the corresponding author.

## Ethics statement

The animal study was reviewed and approved by Animal Experiment Committee of Huazhong Agricultural University (permit number HZAUFI-2016-007).

## Author contributions

QW designed and wrote the main context. MW conducted most experimental protocol. ZC and YW joined the experimental analysis. JZ, SL, and XG conducted the experimental analysis. JG supplied the relevant materials. All authors contributed to the article and approved the submitted version.

## Funding

This article was funded by National Natural Science Foundation of China (Grant No. 32172996; 31802317) and China Scholarship Council (Grant No. CSC[2021]109).

## Conflict of interest

The authors declare that the research was conducted in the absence of any commercial or financial relationships that could be construed as a potential conflict of interest.

## Publisher’s note

All claims expressed in this article are solely those of the authors and do not necessarily represent those of their affiliated organizations, or those of the publisher, the editors and the reviewers. Any product that may be evaluated in this article, or claim that may be made by its manufacturer, is not guaranteed or endorsed by the publisher.
